# Optimal placement of renewable distributed generators and electric vehicles using multi-population evolution whale optimization algorithm

**DOI:** 10.1038/s41598-024-80076-z

**Published:** 2024-11-18

**Authors:** Rinchen Zangmo, Suresh Kumar Sudabattula, Sachin Mishra, Nagaraju Dharavat, Naresh Kumar Golla, Naveen Kumar Sharma, Vinay Kumar Jadoun

**Affiliations:** 1 School of Electronics and Electrical Engineering, Lovely Professional University, Phagwara, 144411 Punjab India; 2 School of Computer Science and Artificial Intelligence, SR University, Warangal, 506371 Telangana India; 3 Department of Electrical and Electronics Engineering, B V Raju Institute of Technology, Telangana, 502313 India; 4 Department of Electrical Engineering, I. K. Gujral Punjab Technical University, Main Campus, Kapurthala, Punjab India; 5 Department of Electrical & Electronics Engineering, Manipal Institute of Technology, Manipal Academy of Higher Education, Manipal, Karnataka India

**Keywords:** Distributed generators, Electric vehicles, Whale optimization algorithm, Power Loss, Voltage Profile, Voltage Stability Index, Renewable energy, Solar energy, Wind energy, Electrical and electronic engineering, Energy grids and networks

## Abstract

This research takes on a crucial task- exploring the optimal placement of Renewable Distributed Generators such as Solar Photovoltaic, wind turbines and Electric Vehicles into the Radial Distribution System (RDS). This is a strategic move aimed at minimising power loss (P_Loss_) and improving the voltage profile and stability index. The RDGs are integrated into RDS with and without considering the uncertainty of the different load demands for 24 h. The probability function of Beta and Weibull distribution functions are employed to attain the solar irradiance and wind speed in a particular region. In addition, EVs are also integrated into RDS, employing meta-heuristic algorithms intended to reduce power loss (PLoss) and improve the voltage profile. The study uses an Indian 28-bus test system mimicking a balanced radial distribution network to integrate distributed generators (DGs) and EV charging stations. The simulated results demonstrate that integrating DGs into power systems has offered considerable benefits, including reduced PLoss, heightened efficiency, decreased dependency on centralised generation, and improved environmental sustainability. It is discovered that the Multi-population Evolution Whale Optimization Algorithm (MEWOA) produces better results than other methods in the literature and is valuable and practical for handling these nonlinear optimisation situations.

**Supplementary Information:**

The online version contains supplementary material available at 10.1038/s41598-024-80076-z.

## Introduction

In the past, electricity production mainly depended on non-renewable resources (coal, oil, and gas) to run thermal facilities using steam turbines and combustion. Hydropower was helpful in areas with plenty of water, and nuclear power was also practical despite safety concerns. However, the depletion of natural resources, their adverse effects on the environment, and the cost of these conventional approaches have forced a paradigm change in favour of renewable energy sources.

In recent times, renewable energy resources (RES) have attracted much attention from researchers because of the promise to provide sustainable and environmentally friendly energy solutions. Compared to non-renewable energy sources that harm the environment and human health, renewable energy sources like solar PV, WT, and EV are better options. A more affordable option to fossil fuels, which are limited resources that worsen the environment, is provided by renewable energy sources. Researchers are developing and improving renewable energy solutions to lessen society’s reliance on non-renewable resources and lessen the adverse consequences of climate change. Development is guided and incentivised by the Paris Agreement’s framework for mitigating climate change and national policy changes, such as India’s 500 GW objective and the EU Green Deal^1^.

However, the advantages mentioned above can only be fully realised if the ideal sizes of DG units are identified and subsequently deployed in the appropriate locations within distribution networks; failure to do so may lead to increased P_Loss_ and voltage deviation. Driven by environmental and electricity demand concerns, numerous strategies have been proposed in the literature to place DGs into the RDS. To harness the optimal benefits of the DGs, it is crucial to place them optimally at a better location. Therefore, various researchers have come up with different types of optimisation techniques. The major goal of power systems is to supply all necessary power while preserving security, dependability, and cost-effective operation. Relying on additional generation sources and strengthening transmission capacity is crucial to addressing problems like the rise in power consumption and environmental concerns^2^. In^3^, an Artificial hummingbird algorithm (AHA) is used to place biomass-based DGs into the three test systems to mitigate the targets of reducing network losses and voltage deviation. In^4^, the authors analysed the impact of integrating DGs using an effective Jellyfish Search Algorithm (JSA) to minimise real P_Loss_ and maximise profits. This article addresses a recent swarm optimisation technique, a backtracking search optimisation algorithm (BSOA), to assign distributed generators (DGs) along radial distribution networks to reduce active and reactive P_Loss_^5^. To minimise P_Loss_ and enhance voltage profile and voltage stability index^6^, suggests a high-convergence optimisation technique for the best placement of distributed generation (DG) in distribution networks. Authors in^7^ describe a novel combination of genetic algorithm (GA)/particle swarm optimisation (PSO) for optimal placement of DGs to reduce network P_Loss_, enhance voltage regulation, and increase voltage stability. The Particle Swarm Optimisation (PSO) algorithm finds DG’s best location and optimal size^8^. Several methods are available in the literature for locating and sizing active and reactive power sources in radial distribution networks^9–14^. The primary goals of these articles are to lower network losses while raising the stability index and voltage profile. It is implemented in various IEEE test systems and verifies the robustness of the algorithms. In^15,16^ The authors aim to minimise total real P_Loss_ and the total voltage deviation to enhance the distribution system performance using an improved genetic algorithm combined with local search (EGA) and salp swarm algorithm (SSA). It is implemented in various test systems. Authors^17^ study the economic benefits of integrating DGs into DS using the PSCAD/EMTDC simulation software package. An effective method is put forth to improve radial distribution networks’ security and dependability and optimise the planning of wind turbine generators and electric vehicle charging. In the IEEE 33-bus distribution test network, the method effectively increased security and reliability indices by sizable percentages^18^.

An RDS that uses renewable energy sources may suffer a significant loss because of the unpredictability of wind and solar irradiance. The intermittent and unpredictable nature of these sources can cause sudden changes in power output, leading to imbalances in the system and increased P_Loss_. Accurate solar irradiance and wind speed prediction are fundamental in managing the P_Loss_ of RDS. Forecasting the potential variations in renewable energy sources provides valuable information to operators so they can plan and adjust power generation accordingly. By doing so, they can minimise P_Loss_ and ensure a stable and reliable power supply. Advanced monitoring and control systems can also be used to manage the power flow and reduce losses in the distribution network.

In conclusion, accounting for the uncertainty associated with solar irradiance and wind speed is critical in managing the P_Loss_ of RDS. It makes system planning and execution more effective while guaranteeing a steady and dependable power supply. Therefore, precise forecasting models and sophisticated monitoring and control systems must be developed to lower uncertainty and boost RDS efficiency. Electric vehicles (EVs) are becoming increasingly ubiquitous; by 2030, there will be over 40 million EVs on the road, drastically changing the nature of electric transportation and posing opportunities and challenges for the power grid^19^. EVs provide better performance and zero carbon emissions, but their integration requires careful thought because of the possibility of grid congestion. Electric vehicles (EVs) are generally known for their benefits to the environment, but integrating them into RDS can be tricky since, if not appropriately placed, they overwhelm the grid. The possible adverse effects of widely integrating electric vehicle charging stations (EVCS), such as voltage deviations, power factor degradation, harmonic distortion, and higher peak load demands, have been noted in several studies^20–22^.

Accordingly, to address the abovementioned problem, numerous research studies were conducted on the design, sizing, and optimal placement of the EVCS in Distribution Systems. In^23^, a novel method based on the Voltage stability, Reliability and P_Loss_ (VRP) index is proposed to place the EV into the distribution system. It is analysed using the IEEE 33 test system. Similarly, the authors of^24–26^ studied the impact of placing the EVs optimally into the distribution system.

The interconnection of EVs increases the peak demand and degrades the voltage profile of the DS. Therefore, it is necessary to schedule the charging of EVs at suitable locations and time intervals. In^27^ authors discuss how to manage the burden of the EVCS by adding the DGs into the distribution systems. Additionally, studies have explored the co-optimisation of EV placement and DG allocation within RDS to minimise P_Loss_, improve voltage profiles, reduce the grid’s stress and maximise EVs’ benefits. The following literature contains a comprehensive analysis of the allocation problem for DGs and EVs: Kumar et al.^28^, Dharavat et al.^29^, Golive et al.^30^, Wang et al.^31^, Mehroliya et al.^32^, CB et al.^33^, Gampa S et al.^34^, Gautam et al.^35^ and Babu et al.^36^. In^37^, a new approach for optimizing the placement of shunt capacitors, electric vehicle charging stations, and distributed generation resources in power distribution networks is introduced. The suggested model integrates fuzzy and chaotic theory with competitive search optimization (CSO) to enhance power efficiency and voltage profiles. Plug in electric vehicles along with PVDG are optimized using the east delta network considering the reduced power loss, reactive power loss and minimum investment cost in^38^.

More studies in the existing literature about the joint optimisation of the architecture of electric vehicle charging stations (EVCS) and distributed generation (DG) deployment, as well as their combined effects on P_Loss_, voltage profiles, and environmental sustainability, need to be conducted This research suggests a novel method for placing DG optimally within RDS to fill this gap. The methodology considers the risks of renewable energy sources, including wind turbines (WT) and solar photovoltaic (PV) systems. It also discusses the best way to distribute EVs and the benefits of combining solar PV and wind turbines with EVCS. The efficacy of the suggested method is assessed using the Indian 28-bus test system and the MEWOA.

The following sums up this paper’s contribution:

Using the MEWOA algorithm, we developed a novel optimisation method for improving the voltage profile and active loss of renewable DG units in RDS.

i. Determine the ideal size or sizes for RDGs and EVs using the suggested MEWOA algorithm.
ii. Determine the solar PV array’s power production and WT for a year or four seasons using the Beta and Weilbull PDF.
iii. Giving input to the suggested algorithms, the actual power output of the solar PV and WT, to determine the real active and reactive loss and how it affects the voltage profile.
iv. When the EV is connected to the RDS, its fixed rating is considered to study its effects on active and reactive P_Loss_ and the RDS’s voltage profile, with the EV acting as the additional load.
v. A system with 28 tests evaluates the suggested algorithm’s robustness and effectiveness.

The best places for distributed generation (DG) are identified using the Voltage Stability Index (VSI), and the sizes of the EV and DG are determined by the MEWOA. The remaining portion of the paper follows the following structure:

- Section “Probabilistic modelling of RDGs” presents the RDGs’ probabilistic models.
- In "Problem Formulation" Sect. , the problem statement is provided.
- The method is described in Sect. 4.
- The results and analysis are discussed in "Results and Discussion" Sect.
- The paper is ended in "Conclusion" Sect.

## Probabilistic modelling of RDGs

Solar and wind power generation is essential to a sustainable energy system. However, because wind speed, ambient temperature, and solar irradiation are all closely correlated with geographic location, their effectiveness heavily depends on these meteorological factors. To guarantee the effective use of photovoltaic (PV) arrays and wind turbines (WTs), it is crucial to thoroughly examine the features of solar radiation and wind conditions at possible installation locations. By paying close attention to details, renewable energy systems may be implemented more intelligently and effectively, leading to a more sustainable and eco-friendly future. When wind and solar generation units are deployed in distributed generation, a more comprehensive uncertainty analysis is necessary due to the load demand uncertainty on the power grid.

### Modeling of solar irradiance

The Beta PDF^39^ estimates the sporadic nature of solar irradiance and is provided below:

1 $$\:{f}_{b}\left(s\right)=\frac{{\Gamma\:}(\alpha\:+\beta\:)}{{\Gamma\:}\left(\alpha\:\right)\:\cdot\:{\Gamma\:}\left(\beta\:\right)}\cdot\:\:{S}^{(\alpha\:-1)}\cdot\:{(1-S)}^{(\beta\:-1)}\:for\:\alpha\:>0;\:\beta\:>0$$

Where.

S is the solar irradiance (kW/m^2^).

$\:{f}_{b}\left(s\right)$ is the Beta distribution function

$\:\alpha\:\:\&\:\beta\:$ are the parameters of the Beta distribution function, and $\:{\Gamma\:}$ represents the Gamma function.

The mean (µ) and standard deviation (σ) of the random variable are used in the following manner to get the parameters of the Beta distribution function [39]:

2 $$\:\beta\:=\:(1-\mu\:)\cdot\:(\frac{\mu\:(1+\mu\:)}{{\sigma\:}^{2}}\:-\:1)$$

3 $$\:\alpha\:=\:\frac{\mu\:\cdot\:\beta\:}{(1-\mu\:)}$$

The power generation of the PV array at solar irradiance (s) for the n^th^ states is evaluated as

4 $$\:{P}_{PVn}\left(S\right)={N}^{PVm}*FF*{V}_{n}\:*{I}_{n}$$

5 $$\:FF=\:\frac{{V}_{MPP}*{I}_{MPP}}{{V}_{OC}*{I}_{SC}}$$

6 $$\:{V}_{n}={V}_{OC}*{K}_{v}*{T}_{cn}$$

7 $$\:{I}_{n}={S[I}_{SC}+{K}_{i}({T}_{C}-25)]$$

8 $$\:{T}_{cn}={T}_{A}+S\left(\frac{{N}_{OT}-20}{0.8}\right)$$

Where $\:{N}^{PVm}$ is the total number of PV modules,

FF is the fill factor.

$\:{T}_{A}$ is the ambient temperature (°C)

$\:{T}_{cn}$ is the cell temperature at nth states

$\:{K}_{v}\:\&\:{K}_{i}$ are the voltage and current temperature co-efficient (A/°C and V/°C)

$\:{N}_{OT}$ is the normal operating temperature of a cell (°C)

$\:{V}_{OC}\:and\:{I}_{SC}$ are the open circuit voltage (V), and short circuit current (A) at maximum power point.

9 $$\:P\left(S\right)\:={P}_{PVn}\left(S\right)*\:{f}_{b}\left(s\right)$$

10 $$\:Total\:Expected\:power\:output\:\left(TEPO\right)=\:\underset{0}{\overset{1}{\int\:}}P\left(S\right)$$

### Modelling of the wind speed

Weibull PDF^39^ is used to estimate the stochastic behaviour of wind speed in a time segment as below:

11 $$\:{f}_{w}\left(\nu\:\right)\:=\frac{K}{C}\cdot\:{\left(\frac{\nu\:}{C}\right)}^{k-1}\cdot\:exp(-({\left(\frac{\nu\:}{C}\right)}^{k-1}\left)\right)\:for\:c>1;\:k>0.$$

Where,

K it the shape parameter and.

C is a scale factor.

The mean (µ) and standard deviation (σ) of the random variable are used in the following manner to get the shape parameter and scale factor of the Weibull distribution function;

12 $$\:k={\left(\frac{\sigma\:}{\mu\:}\right)}^{-1.086}$$

13 $$\:c\:=\frac{\mu\:}{\gamma\:(1+\frac{1}{k})}$$

The power generation of the wind turbine for the nonlinear performance curve for the nth state is provided below equation:

14 $$\:{P}_{WT}\left(\vartheta\:\right)=\:\left\{\begin{array}{c}0\:for\:\:\vartheta\:\le\:{\vartheta\:}^{cut-in}{\:and\:\:\vartheta\:\ge\:\vartheta\:}^{cut-out}\\\:a\cdot\:{\vartheta\:}^{3}+b\cdot\:{P}^{rated}\:for\:{\vartheta\:}^{cut-in}\le\:\vartheta\:\le\:{\vartheta\:}^{rated}\\\:{P}^{rated}\:\:for\:{\vartheta\:}^{rated}\le\:\vartheta\:{\:and\:\:\vartheta\:\ge\:\vartheta\:}^{cut-out}\end{array}\right.$$

15 $$\:a=\frac{{P}^{rated}}{{{\vartheta\:}^{rated}}^{3}-{{\vartheta\:}^{cut-in}}^{3}}$$

16 $$\:b=\frac{{{\vartheta\:}^{cut-in}}^{3}}{{{\vartheta\:}^{rated}}^{3}-{{\vartheta\:}^{cut-in}}^{3}}$$

17 $$\:P\left(WT\right)=\:{P}_{WT}\left(\vartheta\:\right)*\:{f}_{w}\left(\nu\:\right)$$

18 $$\:Total\:Expected\:power\:output\:\left(TEPO\right)=\:\underset{0}{\overset{1}{\int\:}}P\left(WT\right)$$

### Modeling of EV charging station (EVCS)

EVCS is considered the additional load and depends on the charging of the SoC, trip distance and charge power^29^. The SoC of the kth EV at i^th^ hour is given below equation:

19 $$\:{\text{S}\text{o}\text{C}}_{\text{i}}^{\text{k}}=\left(1-\frac{\text{t}\text{*}\text{d}}{{\text{d}}_{\text{c}}}\right)\cdot\:100\text{\%}$$

Battery Storage constraints of the EV is given by:

20 $$\:{SoC}^{min}\le\:{SoC}_{i}^{EV}\le\:{SoC}^{max}$$

21 $$\:{P}_{i,d}^{ch}\le\:{P}_{max,\:i}^{ch}$$

22 $$\:{P}_{i,d}^{disch}\le\:{P}_{max,\:i}^{disch}$$

Where,

t is the no. of trips, d is the distance travelled by EV, $\:{d}_{c}$ is the distance covered by EV in the range

### Problem formulation

The unique methodology focuses primarily on maximising P_Loss_ reduction and voltage profile improvement by allocating solar, wind, and electric vehicles (EVs) in the RDS in the best possible way. The overall real P_Loss_ is expressed below:

23 $$\:min\sum\:_{b=1}^{24}{P}_{LOSS}$$

24 $$\:{P}_{LOSS}=\sum\:_{b=1}^{24}\left({I}^{2}*{R}_{b}\right)$$

The bus voltage should be maintained within the permissible limits and power generation should be equal to the power demand and P_Loss_ in the RDS. The voltage and power constraints are provided below:

25 $$\:{V}_{min}^{b,\:i}\le\:{V}_{b,\:i}\le\:{V}_{max}^{b,i}$$

26 $$\:{P}_{DG}^{min}\le\:{P}_{DG}\le\:{P}_{DG}^{max}$$

27 $$\:\sum\:_{b=1}^{24}{P}_{G}^{SS}+\sum\:_{b=1}^{24}{P}_{DG}^{PV}+\sum\:_{b=1}^{24}{P}_{DG}^{WT}={\sum\:}_{b=1}^{24}\left[{P}_{Demand}+{P}_{EV}+{P}_{Loss}\right]$$

Where,

$\:{P}_{G}^{SS}$ is power generated from the grid substation,

$\:{P}_{DG}^{PV}$ is power generated by solar PV DGs

$\:{P}_{DG}^{WT}$ is power generated by WT DGs

$\:{P}_{Demand}$ is the total demand of the system

$\:{P}_{EV}$ is the power consumed by the EV while charging

$\:{P}_{Loss}$ total system losses.

## Voltage stability index (VSI)

**Fig. 1:**
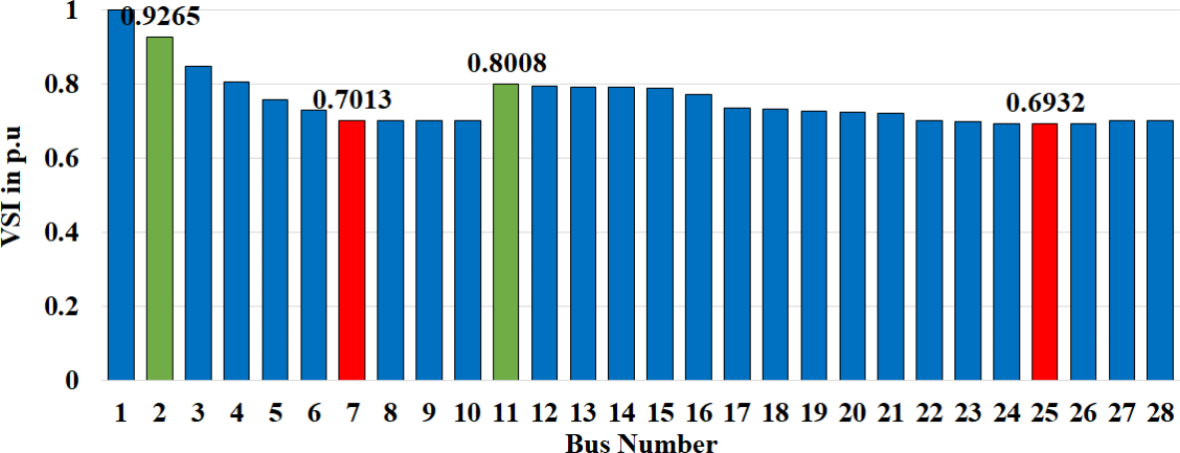
The VSI value at the base case (without DG and EV) shows the weak and strong buses.

The voltage stability indicator (VSI)^40^, is a vital power system tool. It helps to understand the probability of voltage collapse and identify weak or strong buses in the system. Additionally, the VSI can help find the best places to distribute energy resources (DERs). Using the VSI, power system operators can better decide where to put DERs and how to improve their system’s voltage stability. The bus is stronger if the VSI value is closer to 1, and the weak bus is if the VSI value is closer to 0. Figure 1represents the VSI value of each bus without considering any DG and EV. The red column indicates the weak bus, and the green column indicates the stronger bus, according to the VSI. The solar PV and WT will be placed in the weaker buses, and EVs will be placed in the stronger buses identified by VSI. VSI is calculated from the load flow solution data as given in the equation below^41^:

28 $$\:VSI=2{V}_{1}^{2}{V}_{2}^{2}-{V}_{2}^{4}-{2 V}_{2}^{2}\left(PR+QX\right)-\left({P}^{2}+{Q}^{2}\right){\left|Z\right|}^{2}$$

## Optimisation algorithm

**Fig. 2:**
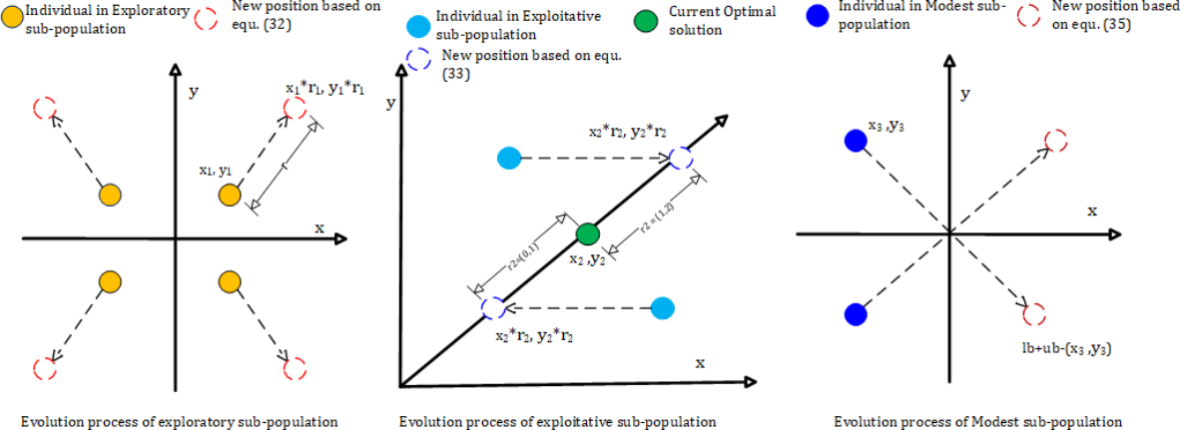
Evolution strategy of MEWOA.

WOA is an optimisation technique developed by Mirjalili S. in 2019 to solve single and multi-objective optimisation problems in various fields. However, WOA frequently experiences rapid local optimum and delayed convergence and may become trapped in undesirable outcomes. Additionally, it can be challenging to investigate the entire solution space efficiently. Therefore, Ya Shen et al. developed improved versions of the WOA addressing these shortcomings in 2022. MEWOA unveils a unique feature based on the evolution of multi-population and is a vital tool for optimising complicated problems in power systems, such as integrating PV, WTs, and EVs into RDS. MEWOA imitates humpback whale hunting techniques, such as encircling prey to investigate it and using bubble nets to harvest it with exploration and exploitation methods. The MEWOA algorithm groups the whale population into three sub-populations based on their fitness levels. Individuals with poor fitness levels are categorised as exploratory sub-populations, those with good fitness levels as exploitative sub-populations, and those in between, good and bad, are classified as modest sub-populations. Each sub-population consists of an equal number of individuals and follows a distinct moving strategy, as shown in Fig. 2.

Each moving strategy of each sub-population can be represented using the following equations:

i. Exploratory sub-population.

The movement of the whale moving randomly to explore globally is represented by Eq. 6, when the |A|>1;

29 $$\:X(t+1)={X}^{rand}\left(t\right)-A\cdot\:|C\cdot\:{X}^{rand}(t)-X(t\left)\right|$$

Where: $\:{X}^{rand}$ is the random whale picked from the current population at the $\:{t}^{th}$ iteration. |A| is the absolute value of A. X(t) is a whale whose position vector needs to be updated. A and C are the coefficient random vectors between (0,1).

ii. Exploitative sub-population.

Usually, the whales swim in the decreasing circle of the prey, so there is a 50% probability of catching the prey and a 50% possibility of not getting the prey. Therefore, this process can be represented by Eq. 5.

30 $$\:X(t+1)=\left\{\begin{array}{c}X*\left(t\right)-A\cdot\:|C\cdot\:X*(t)-X(t\left)\right|\:\:if\:p<0.5\\\:\left|X*\right(t)-X(t\left)\right|\cdot\:{e}^{bl}\cdot\:cos\:\left(2\pi\:l\right)+X*\left(t\right)\:if\:p\ge\:0.5\end{array}\right.$$

Where X*(t) is the position vector of the best whale, p is the random vector between (0,1). b is the constant number usually set at 1, and l is the random number between [−1,1].

iii. Modest sub-population.

31 $$\:X(t+1)=\left\{\begin{array}{c}\left\{\begin{array}{c}X*\left(t\right)-A\cdot\:|C\cdot\:X*(t)-X(t\left)\right|\:\:if\:{p}_{1}<0.5\\\:\left|X*\right(t)-X(t\left)\right|\cdot\:{e}^{bl}\cdot\:cos\:\left(2\pi\:l\right)+X*\left(t\right)\:if\:{p}_{1}\ge\:0.5\end{array}\right.\:if\:{p}_{2}<0.5\\\:{X}^{rand}\left(t\right)-A\cdot\:|C\cdot\:{X}^{rand}(t)-X(t\left)\right|\:if\:{p}_{2}\ge\:0.5\end{array}\right.$$

Once the positions of the individuals are updated, with every iteration, the population evolution is also considered to increase the population’s search diversity, escape local optima, and accelerate convergence. The following equations represent the population evolution of the sub-populations.

i. Exploratory sub-population.

32 $$\:X(t+1)=X\left(t\right)\cdot\:{r}_{1}$$

Where $\:{r}_{1}$ is the random number between (1,2).

ii. Exploitative sub-population.

33 $$\:X(t+1)={X}^{*}\left(t\right)\cdot\:{r}_{2}$$

Where $\:{r}_{2}$ is the random number between (0,2) and $\:{X}^{*}\left(t\right)$ is the position vector of a present optimal solution.

iii. Modest sub-population.

34 $$X(t+1) = \left\{ \begin{array}{ll} X^{(t)}\, if\, fit (X^{(t)})\, is\, better\, then\, fit (X(t))\\ X(t)\, else \end{array} \right.$$

35 $$\:X^{(t)}=lb+ub-X(t)$$

Where $\:X^{(t)}$ is the new position of the objective function, lb is the lower boundary, ub is the upper boundary and fit () is the objective function of the problem.

The sequence of steps of MEWOA is provided as follows:

Step 1. Read the system data of the proposed test system.

Step 2. Run the Backward Forward DLF.

Step 3. Using the VSI method, calculate the VSI of each bus.

Step 4. Identify the weak and strong buses for allocating DGs and EVs, respectively. Accordingly, the information will be provided to the MEWOA algorithm.

Step 5. Initialize the parameters of the MEWOA (population sizes, maximum iteration, lower bound, upper bound, and the dimension of the fitness function, and set the current iteration to 1).

Step 6. Compute the fitness of all individuals and select the best fitness values as the current optimal solution.

Step 7. Divide the population into three sub-populations based on fitness levels: exploratory, exploitative, and modest.

Step 8. According to the current iteration number, update the position as per Eqs. 29, 30, and 31 if the iteration is odd and update the population as per Eqs. 32, 33, and 34 if it is even.

Step 9. Then, update the fitness values (P_Loss_).

**Fig. 3:**
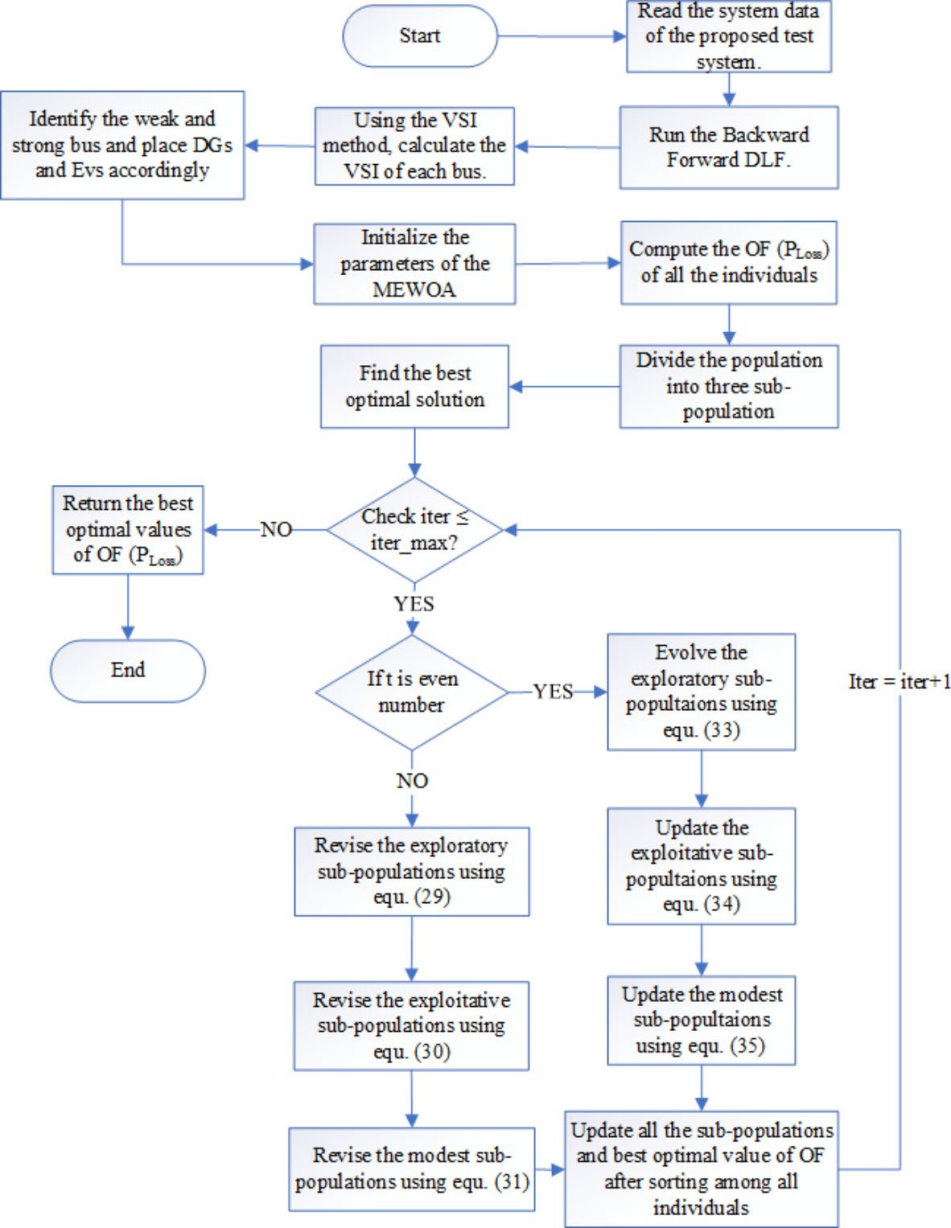
Flow chart of MEWOA.

The MEWOA flow chart is represented in Fig. 3.

## Results and discussion

**Fig. 4:**
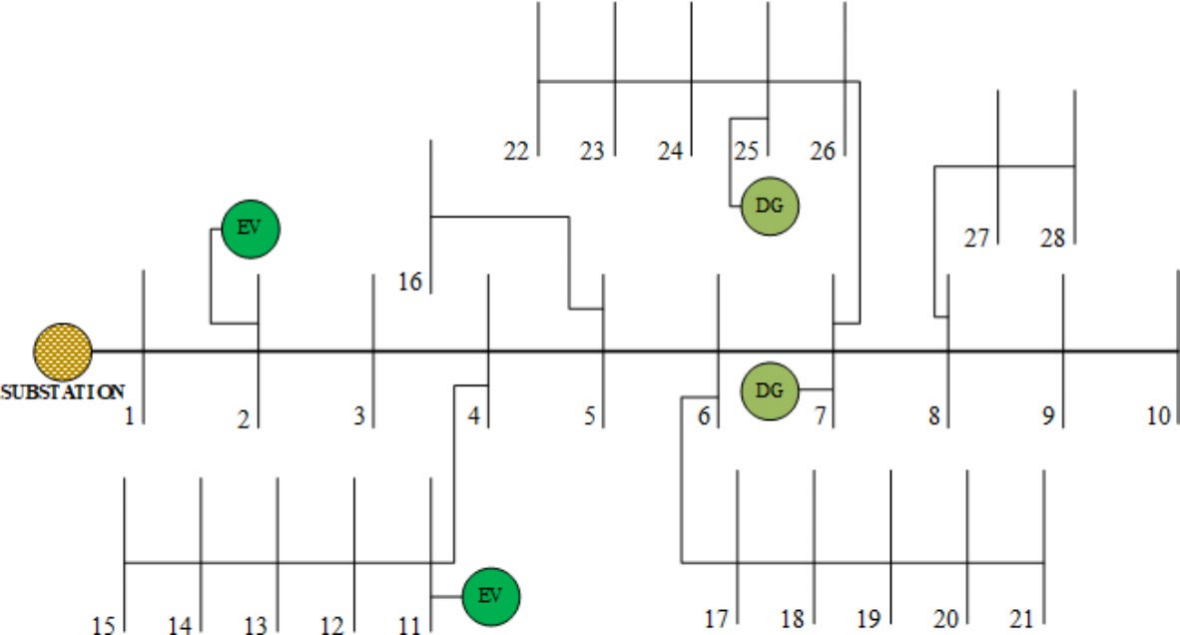
Single-line diagram of 28 test systems with two DGs and two EVs.

This section describes implementing the proposed technique in the Indian 28 test system. Figure 4 depicts the single-line diagram of the 28-bus test system, which consists of 27 branches and 28 buses operating at a base voltage of 11 kV. The system’s total demand load is 776.42 kVar and 761.04 kW. The main objectives of this paper are to optimise the goal functions of the RDS, which include minimising P_Loss_, improving the voltage profile, and maximising VSI. Several possibilities are examined in this work in the following ways:

i. Effects of RDGs on system losses and voltage profile.
ii. Solar PV and WT affect system losses and voltage profile considering uncertainty for different seasons.
iii. Effects of EVs on system losses and voltage profile without DGs.
iv. Effects of DGs and EVs on system losses and voltage profile.

## Effects of RDGs on system losses and voltage profile

The suggested method was first implemented and evaluated for the base case, which uses the standard line and bus data of the 28-test system to perform the backwards-forward distribution load flow (DLF) to obtain the active P_Loss_. This article also simulates the various load profiles 24 h after integrating one DG and two DGs into the RDS. Table 1 lists the simulated outcomes of the base case, one DG and two DGs active and reactive P_Loss_. As per the findings presented in Table 1, it is evident that the P_Loss_ experienced by the system is directly proportional to the load demand of the customers. At full load demand (at 18 h), the active P_Loss_ reaches its maximum value of 68.8189 kW, and the reactive P_Loss_ reaches its maximum value of 46.0416 kVar. However, at 50% load demand (4 & 5 h), the active P_Loss_ is 21.751 kW, and the reactive P_Loss_ is 14.55 kVar.

**Fig. 5:**
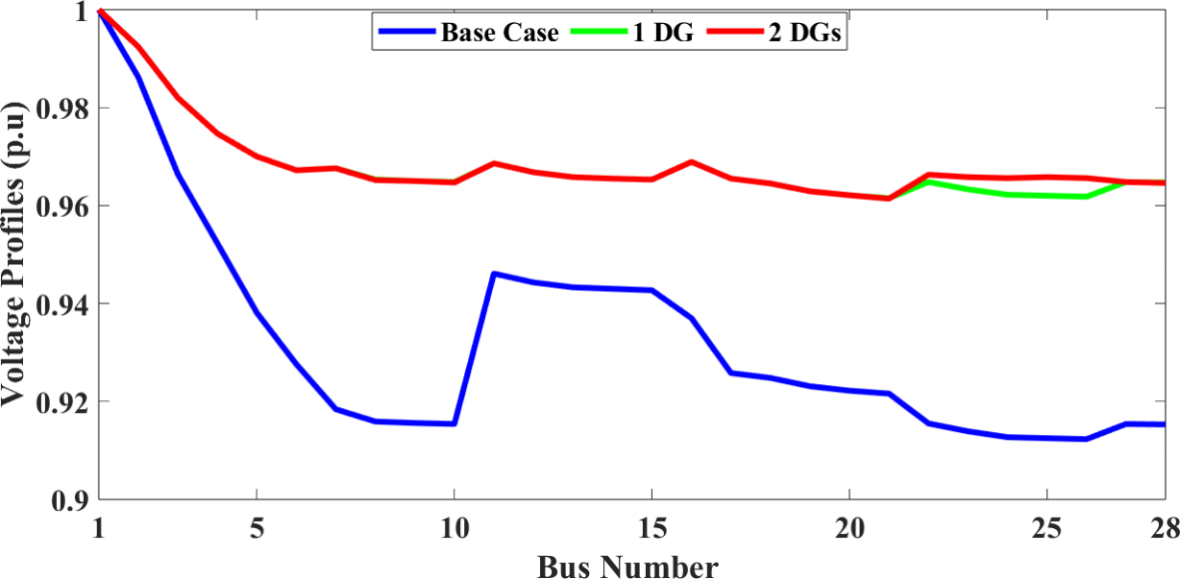
Voltage profile of the 28-bus system with different scenarios.

**Fig. 6:**
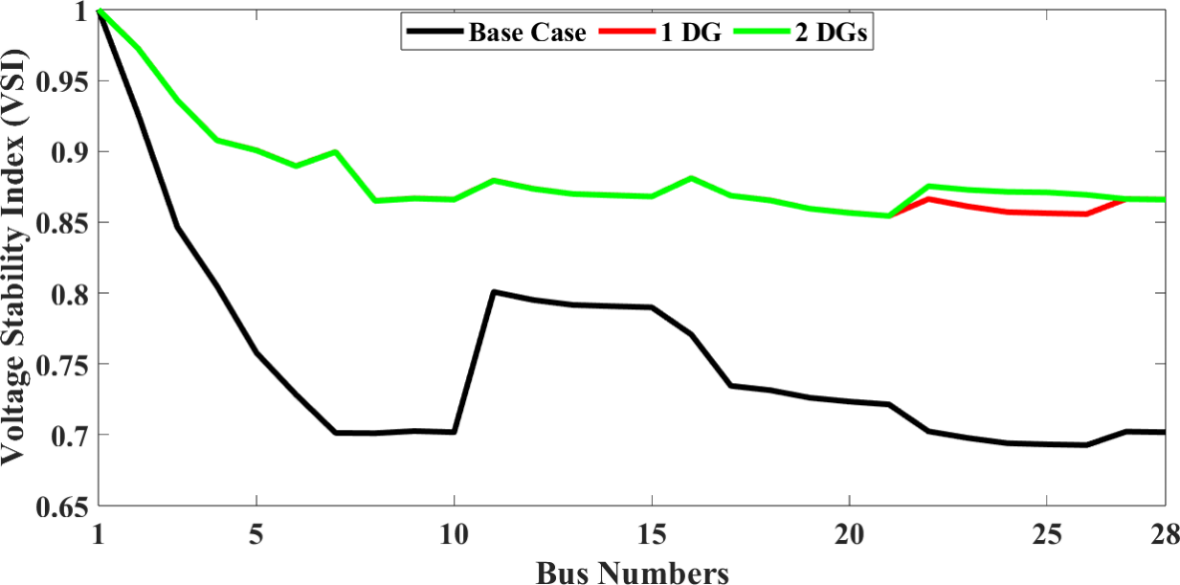
Voltage stability index (VSI) of the 28-bus system with different scenarios.

To validate the objective of reducing P_Loss_ and enhancing the voltage profile, a distributed generator (DG) was incorporated at bus location 7 in the RDS. The results of this addition revealed a significant decrease in active P_Loss_ to 37.0066 kW and reactive P_Loss_ to 24.3207 kVar at full load demand (18 h) and active P_Loss_ to 12.1178 kW and reactive P_Loss_ to 7.9626 kVar at 50% load demand. Further, two DGs are added into the RDS, and the active and reactive P_Loss_ is further reduced to 36.53 kW and 24.12 kVar, respectively, for full load demand. Therefore, adding DGs can significantly lower the P_Loss_ and raise overall voltage profiles and VSI. The effects of DGs on voltage profile and VSI are graphically represented in Figs. 5 and 6, respectively.

**Table 1:** Analysis of active/reactive P_Loss_ in DN: base case vs. single/double DG scenarios.

Hours	Demand	Base Case		1 DG				2 DGs				
		Active *P*_Loss_	Reactive *P*_Loss_	Active *P*_Loss_	Reactive *P*_Loss_	DG Size	VI_min_	Active *P*_Loss_	Reactive *P*_Loss_	DGs Size		VI_min_
1	0.661	28.4096	19.0046	15.7289	10.3357	379.73	0.9749	15.53	10.25	304.19	75.14	0.9749
2	0.621	24.9163	16.6676	13.8388	9.0936	356.15	0.9765	13.66	9.02	290.45	65.02	0.9765
3	0.604	23.5076	15.7251	13.0739	8.5909	346.15	0.9771	12.91	8.52	277.17	68.64	0.9771
4	0.582	21.751	14.55	12.1178	7.9626	333.23	0.9780	11.96	7.90	266.78	66.14	0.9780
5	0.582	21.751	14.55	12.1178	7.9626	333.23	0.9780	11.96	7.90	266.78	66.14	0.9780
6	0.600	23.1827	15.5078	12.8973	8.4748	343.80	0.9773	12.73	8.41	275.28	68.19	0.9773
7	0.750	37.1053	24.8223	20.3944	13.4019	432.52	0.9714	20.13	13.29	346.71	85.28	0.9714
8	0.864	50.1845	33.5732	27.317	17.9518	500 ~ 0.75	0.9669	26.96	17.80	401.89	98.17	0.9669
9	0.946	61.0107	40.817	32.9697	21.6672	550.28	0.9636	32.54	21.49	441.78	107.64	0.9636
10	0.957	62.5568	41.8515	33.7717	22.1943	556.95	0.9632	33.33	22.01	447.17	108.90	0.9632
11	0.954	62.1329	41.5679	33.5519	22.0499	555.13	0.9633	33.12	21.86	445.70	108.56	0.9633
12	0.946	61.0107	40.817	32.9697	21.6672	550.28	0.9636	32.54	21.49	441.78	107.64	0.9636
13	0.939	60.0386	40.1665	32.4648	21.3354	546.03	0.9639	32.04	21.16	438.35	106.84	0.9639
14	0.957	62.5568	41.8515	33.7717	22.1943	556.95	0.9632	33.33	22.01	447.17	108.90	0.9632
15	0.929	58.6655	39.2478	31.7508	20.866	539.98	0.9643	31.34	20.69	433.45	105.70	0.9643
16	0.939	60.0386	40.1665	32.4648	21.3354	546.03	0.9639	32.04	21.16	438.35	106.84	0.9639
17	0.982	66.1549	44.2591	35.6329	23.4178	572.14	0.9622	35.17	23.22	459.46	111.76	0.9622
18	1.000	68.8189	46.0416	37.0066	24.3207	583.10	0.9615	36.53	24.12	468.32	113.81	0.9614
19	1.000	68.8189	46.0416	37.0066	24.3207	583.10	0.9615	36.53	24.12	468.32	113.81	0.9614
20	0.957	62.5568	41.8515	33.7717	22.1943	556.95	0.9632	33.33	22.01	447.17	108.90	0.9632
21	0.911	56.2403	37.6251	30.4871	20.04	529.09	0.9650	30.09	19.87	424.66	103.65	0.9650
22	0.829	45.9301	30.7266	25.077	16.4795	479.73	0.9683	24.75	16.34	384.79	94.29	0.9683
23	0.725	34.5317	23.1005	19.0192	12.4981	417.65	0.9724	18.78	12.39	334.73	82.43	0.9724
24	0.625	25.2543	16.8937	14.0221	9.214	358.50	0.9763	13.84	9.14	287.11	71.04	0.9763

**Solar PV and WT affect system losses and voltage profile considering uncertainty for different seasons.**

**Fig. 7:**
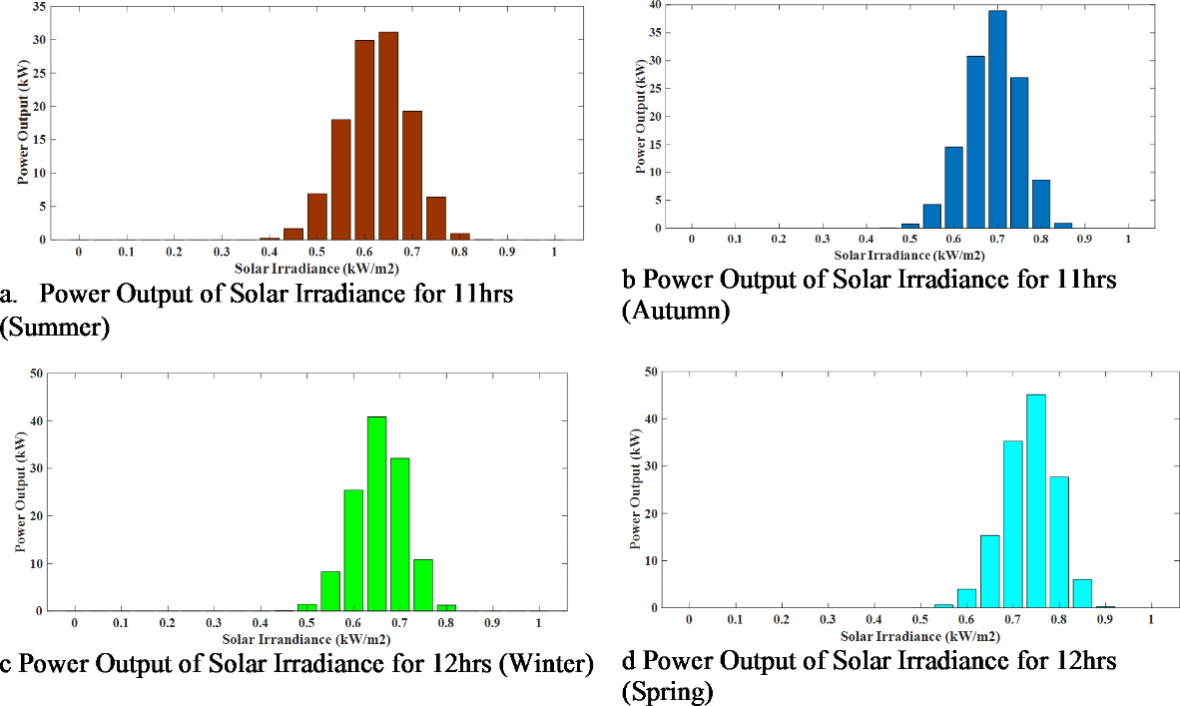
Power output of solar irradiance for four seasons.

A 250 kW WT and a 600 PV module 132 kW Solar PV array are used to precisely analyse wind and solar power’s erratic and intermittent characteristics. The Solar PV and WT specifications are provided in Tables 2 and 3, respectively. This study chose a year as the study period, which was divided into four seasons. A typical day is generated for each season to reflect the random behaviour of the various renewable resources over time. It is standard procedure to employ probability density functions (PDFs) to estimate the hourly wind speed and solar irradiance data in this investigation. The Weibull and Beta distributions are two popular PDFs. These distributions are often discretised by dividing the data into a predetermined number of states. In this case, the number of states chosen for the Beta and Weibull PDFs were 20 and 15, respectively. The distribution of hourly wind speed and solar irradiance at the research location can be more precisely modelled and analysed by discretising the data in this way. The power output of solar irradiance at 11 h during summer and autumn and 12 h during winter and spring has been graphically represented in Fig. 7.

**Fig. 8:**
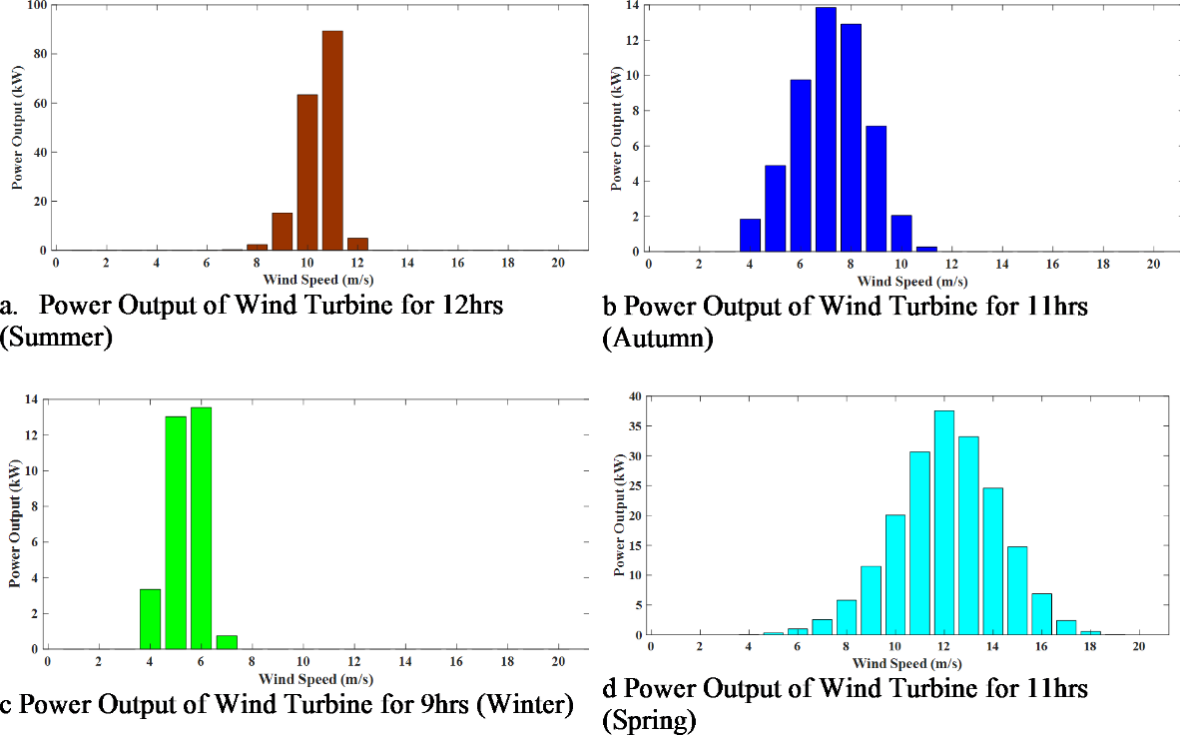
Power output of wind speed for four seasons.

**Fig. 9:**
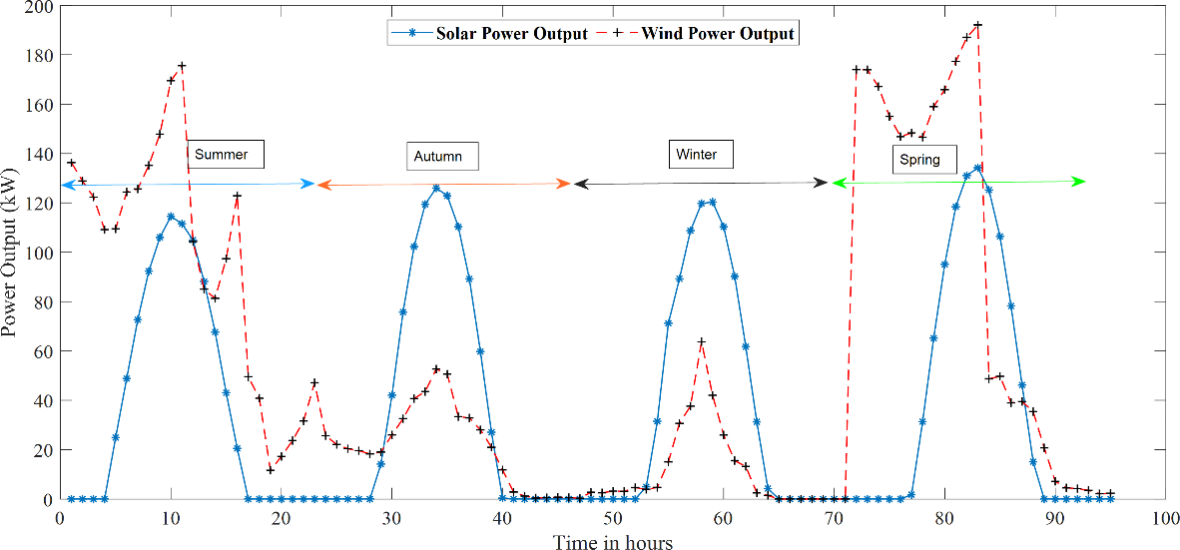
Hourly power output of solar and wind for four different seasons.

Similarly, Fig. 8 depicts the power output of wind speed for specific hours during the four seasons. These representations provide a comprehensive and visual understanding of the power output fluctuations of both solar irradiance and wind speed throughout the year. The summarised power output generated by the solar irradiance and wind speed are illustrated in Fig. 9. The total of 96-time segments representing four seasons is depicted in Fig. 9. It can be seen that solar irradiance is maximum during noon for all four seasons, and wind speed is maximum during the spring and summer seasons.

**Table 2:** Specification of Solar PV^39^.

Parameter	Value
The voltage at the maximum power point, V_MPP_	28.36 V
The voltage at the maximum power point, I_MPP_	7.76 A
Open circuit voltage, V_oc_	36.96 V
Short circuit current, I_sc_	8.38 A
Nominal cell operating temperature, N_OT_	43 °C
Current temperature coefficient	0.00545 A/ °C
Voltage temperature coefficient	0.1278 V/ °C

**Table 3:** Specification of WT^39^.

Parameters	Value
Rated output power, Prated	250 kW
Cut-in-speed, v_cin_	3 m/s
Nominal wind speed, V_N_	12 m/s
Cut-out speed, v_cout_	25 m/s

After obtaining the actual power output of the solar photovoltaic (PV) and wind turbine (WT), their outputs are incorporated into a 28-test system to investigate the impacts of these distributed generators (DGs) on P_Loss_ and voltage profiles. Initially, a single solar PV with a rating of 132 kW is connected to the 28-test system at bus location 7. Subsequently, two solar PVs are connected at bus locations 7 and 25, respectively, to study the effects of the uncertainty of solar irradiance. The results obtained from the experiments are carefully tabulated in Table 4. The methodology used in this investigation offers empirical evidence that integrating single and two Solar PVs results in a slight decrease in P_Loss_, specifically to 57.0926 kW and 47.6817 kW during the 11 h in summer. It should be noted that this methodology has applicability across all seasons, given that the solar irradiance is at its maximum during the 11 h across all seasons. These findings underscore the potential of Solar PVs as a viable solution to address P_Loss_, highlighting the significance of considering seasonal variations in solar irradiance when designing solar PV systems.

**Table 4:** Comparison of active P_Loss_ for integrating single SPV and two SPV DGs to the DN considering the uncertainty of solar irradiance for four seasons.

Hour	Summer		Autumn		Winter		Spring	
	1 SPV	2 SPV	1 SPV	2 SPV	1 SPV	2 SPV	1 SPV	2 SPV
1	0	0	0	0	0	0	0	0
2	0	0	0	0	0	0	0	0
3	0	0	0	0	0	0	0	0
4	0	0	0	0	0	0	0	0
5	68.8183	68.8176	0	0	0	0	0	0
6	66.0106	63.1162	67.2036	65.4962	68.2494	67.6346	68.6118	68.3866
7	63.4538	58.2423	64.1641	59.5641	65.2952	61.7206	65.31	61.7492
8	61.0366	53.9449	60.7345	53.4309	61.1793	54.1897	61.7844	55.2402
9	59.1387	50.8062	58.2073	49.3487	59.4293	51.2724	58.8769	50.3907
10	57.8652	48.8279	56.6603	47.0589	57.6145	48.4513	56.7479	47.184
11	57.0926	47.6817	56.0773	46.2412	56.627	47.0115	55.6426	45.6486
12	**57.3634**	48.0786	56.3515	46.6225	56.5768	46.9402	55.3718	45.2868
13	57.9635	48.9767	57.4708	48.2375	57.4722	48.2396	56.1461	46.3363
14	59.5333	51.4406	59.4345	51.2809	59.3386	51.1264	57.8263	48.7692
15	61.5315	54.7986	62.3297	56.2044	62.1265	55.8432	60.4912	53.0207
16	64.0614	59.3714	65.7798	62.6634	65.3202	61.7689	63.7345	58.7616
17	66.4977	64.0802	68.7716	68.7201	68.3247	67.7904	67.1013	65.2896
18	0	0	0	0	0	0	0	0
19	0	0	0	0	0	0	0	0
20	0	0	0	0	0	0	0	0
21	0	0	0	0	0	0	0	0
22	0	0	0	0	0	0	0	0
23	0	0	0	0	0	0	0	0
24	0	0	0	0	0	0	0	0

Like solar PVs, wind turbines are integrated into the 28-test system to examine their effect on power system stability. To begin with, a 250 kW-rated WT is linked at bus location 7. Following that, two wind turbines are connected at bus locations 7 and 25 to analyse how wind speed unpredictability affects the system. The outcomes from these trials are meticulously documented in Table 5. According to the data in Table 5, the wind speed reaches its highest during spring, specifically at noon, resulting in a PLoss of 40.1782 kW.

**Fig. 10:**
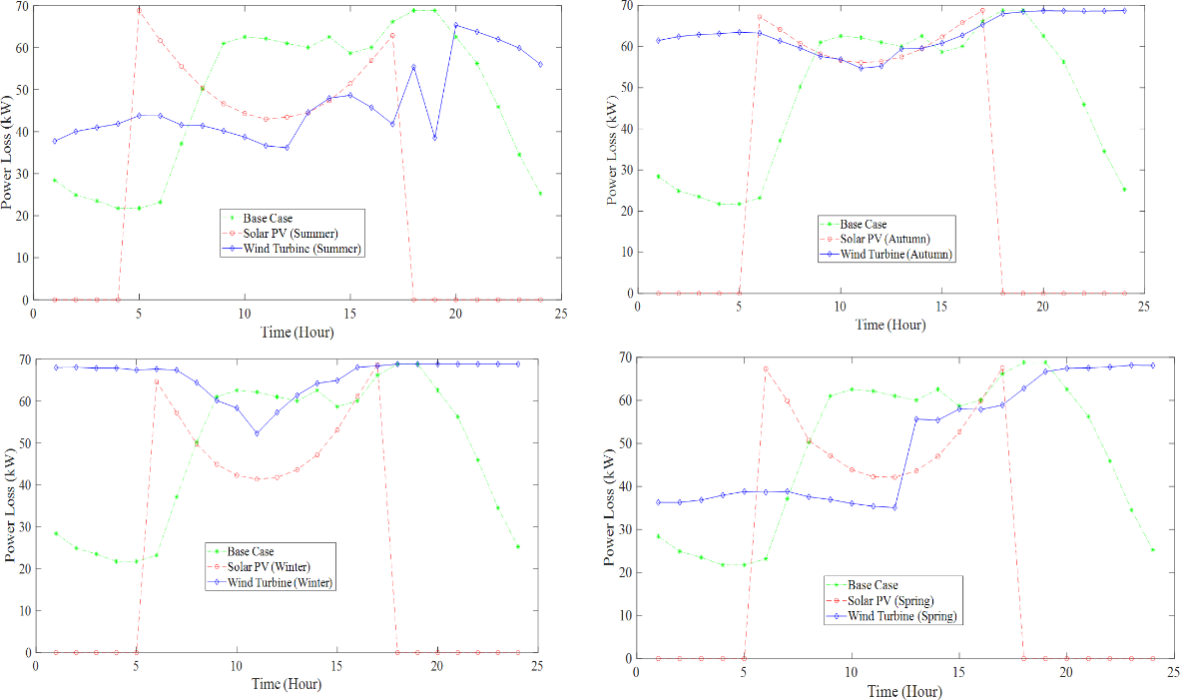
Hourly P_Loss_ of Solar and Wind for four seasons (Summer, Autumn, Winter, and Spring) considering uncertainty.

**Fig. 11:**
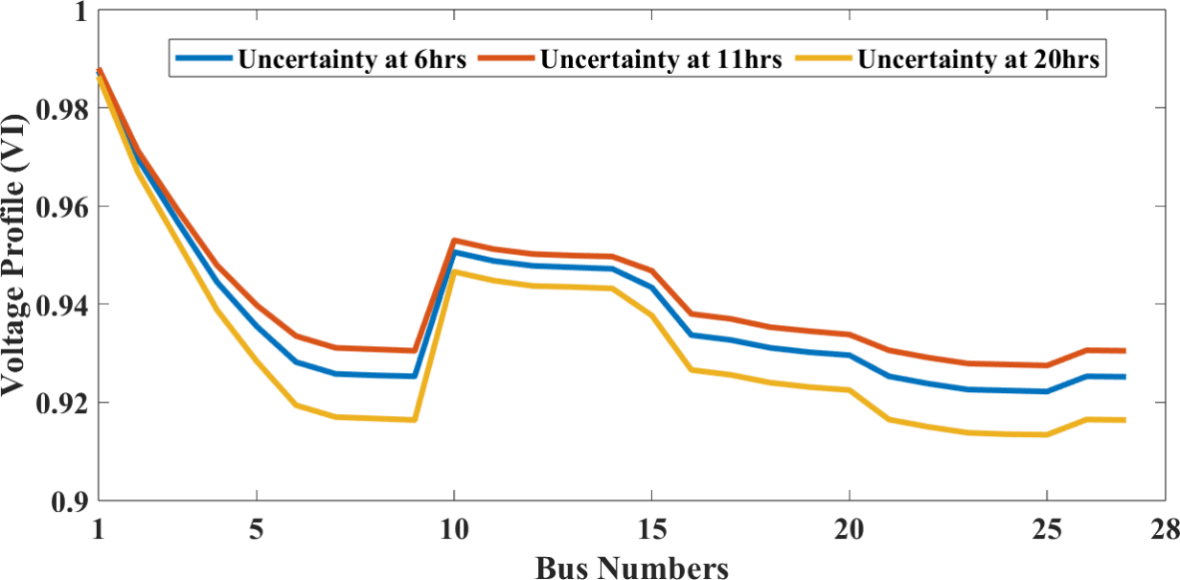
Voltage profile graph of 1 WT integration considering the wind speed uncertainty at three different times (summer).

Conversely, wind speeds are noticeably lower during autumn, resulting in a corresponding decrease in PLoss. The presented study focuses on the effects that wind turbine and solar PV penetration have on RDS concerning PLoss and voltage profiles during different seasons. Figure 10 illustrates the different scenarios of PLoss. The voltage profiles at three different times, considering the uncertainty of WT, are represented in Fig. 11. The figures indicate that the voltage profiles vary according to their power output.

**Table 5:** Comparison of active P_Loss_ for integrating single WT and two WT DGs to the DN considering the wind speed uncertainty for four seasons.

Hour	Summer		Autumn		Winter		Spring	
	1 WT	2 WT	1 WT	2 WT	1 WT	2 WT	1 WT	2 WT
1	53.458	42.9072	65.9312	62.9601	68.5004	68.1548	52.1449	41.4699
2	55.1931	45.0514	66.3228	63.7327	68.5259	68.2079	52.1515	41.4767
3	55.8286	45.9003	66.5118	64.1083	68.4491	68.0483	52.679	42.0339
4	56.401	46.6921	66.5995	64.2831	68.46	68.0709	53.6428	43.1229
5	57.5777	48.3964	66.7476	64.5789	68.2828	67.7038	54.3111	43.9285
6	57.5495	48.3544	66.6617	64.4072	68.3677	67.8795	54.1883	43.7775
7	56.2192	46.4378	65.8939	62.887	68.2759	67.6894	54.3337	43.9565
8	56.1135	46.2912	65.1821	61.5021	67.1034	65.294	53.3246	42.7535
9	55.2876	45.1757	64.3114	59.8413	65.386	61.8963	52.7781	42.1417
10	54.2279	43.8261	64.0117	59.2784	64.6348	60.4539	51.8964	41.2176
11	52.4934	41.8346	**63.0571**	**57.5154**	**61.9292**	**55.4946**	51.1623	40.5112
12	**52.024**	**41.3464**	63.2705	57.9056	64.1697	59.5745	**50.7923**	**40.1782**
13	**58.024**	49.0686	65.0943	61.333	65.8998	62.8986	63.4681	58.2686
14	59.8329	51.9288	65.1499	61.4401	67.0502	65.1865	63.363	58.0753
15	60.1915	52.5203	65.6708	62.4503	67.3119	65.7154	64.4928	60.1843
16	58.6617	50.0527	66.4455	63.9764	68.5275	68.2112	64.4392	60.0828
17	56.3505	46.6212	67.4683	66.0329	68.6496	68.4654	64.8734	60.9091
18	63.383	58.112	68.4915	68.1364	68.8188	68.8187	66.4722	64.0294
19	64.2947	59.8098	68.6773	68.5232	68.8189	68.8189	67.9969	67.1139
20	67.4872	66.0713	68.7698	68.7162	68.8189	68.8189	68.2969	67.7329
21	66.8588	64.8018	68.7528	68.6808	68.8189	68.8189	68.3312	67.804
22	66.1478	63.3867	68.7347	68.643	68.8189	68.8189	68.4141	67.9757
23	65.2848	61.7004	68.7466	68.6678	68.8189	68.8189	68.57	68.2996
24	63.6355	58.578	68.7792	68.7359	68.8189	68.8189	68.5475	68.2529

Significant values are in bold.

### Effects of EVs on system losses and voltage profile without DGs

Reducing carbon emissions and supporting sustainable mobility are two reasons the distribution system’s integration of electric vehicles (EVs) has attracted much interest. However, the integration of these vehicles can have a significant impact on P_Loss_ and voltage profile within the RDS. Therefore, EV charging stations are connected to the RDS at different bus locations identified by VSI to verify its effects on RDS. This paper uses the proposed algorithm to study the effects on P_Loss_ and voltage profile. The detailed specifications for EVs are provided in Table 6. Using the specification provided in Table 6, the rating of the EV charging station is calculated as 142 kW. One EV charging station is initially connected to charge at bus location 2. Subsequently, two EV charging stations are connected to the RDS at bus locations 2 and 11. The simulated results of P_Loss_ and voltage profile for 24 h are tabulated in Table 7. With the addition of the EV, the load demand in the RDS increases, and based on the rating of the EV charging station, the P_Loss_ increases slightly based on the power consumed by the EV while charging. From Table 7, results clearly show that with the addition of one EV at location 2, the P_Loss_ increased to 70.6467 kW from 68.8195 kW, and when two EVs were added to the RDS at 2 and 11, P_Loss_ increased to 84.3528 kW from 68.8195 kW. However, the voltage profile is decreased to 0.9108 (p.u) and 0.9048 (p.u) from 0.9123 (p.u) with the addition of single and two EVs into RDS, respectively.

**Table 6:** Specification of EV^29^.

Parameters	Value
EV Capacity or Battery	16 kWh
No. of EVs	60
SoC min	0.2
SoC max	0.9
Average power consumption per km	0.175kWh/km
Average distance travelled by each EV	30 km

**Table 7:** Active and reactive P_Loss_ with integration of EVs.

	Base Case			1 EV @ 2			2 EV @ 2 & 11		
Demand	Active *P*_Loss_	Reactive *P*_Loss_	Vmin	Active *P*_Loss_	Reactive *P*_Loss_	Vmin	Active *P*_Loss_	Reactive *P*_Loss_	Vmin
**0.661**	28.4098	19.0048	0.9438	30.2113	20.2374	0.9423	38.579	25.5963	0.9367
**0.621**	24.9165	16.6677	0.9474	26.6091	17.826	0.9459	34.509	22.8808	0.9403
**0.604**	23.5078	15.7253	0.9489	25.1548	16.8524	0.9474	32.8585	21.7797	0.9418
**0.582**	21.7512	14.5501	0.9508	23.3395	15.6371	0.9493	30.7918	20.401	0.9438
**0.582**	21.7512	14.5501	0.9508	23.3395	15.6371	0.9493	30.7918	20.401	0.9438
**0.600**	23.1828	15.5079	0.9492	24.8191	16.6277	0.9477	32.477	21.5251	0.9422
**0.750**	37.1056	24.8225	0.9357	39.1557	26.225	0.9342	48.5973	32.2824	0.9285
**0.864**	50.1849	33.5734	0.9252	52.5676	35.2031	0.9237	63.4563	42.2023	0.9179
**0.946**	61.0112	40.8173	0.9175	63.6438	42.6175	0.9159	75.6273	50.3298	0.9101
**0.957**	62.5573	41.8519	0.9164	65.2241	43.6754	0.9149	77.3581	51.4858	0.909
**0.954**	62.1334	41.5682	0.9167	64.7909	43.3854	0.9152	76.8837	51.1689	0.9093
**0.946**	61.0112	40.8173	0.9175	63.6438	42.6175	0.9159	75.6273	50.3298	0.9101
**0.939**	60.0391	40.1669	0.9181	62.6499	41.9523	0.9166	74.5381	49.6024	0.9107
**0.957**	62.5573	41.8519	0.9164	65.2241	43.6754	0.9149	77.3581	51.4858	0.909
**0.929**	58.666	39.2481	0.9191	61.246	41.0124	0.9175	72.9986	48.5743	0.9117
**0.939**	60.0391	40.1669	0.9181	62.6499	41.9523	0.9166	74.5381	49.6024	0.9107
**0.982**	66.1555	44.2595	0.914	68.9008	46.1366	0.9125	81.3802	57.1205	0.9066
**1.000**	**68.8195**	**46.042**	**0.9123**	**70.6467**	**47.2913**	**0.9108**	**84.3528**	**56.1575**	**0.9048**
**1.000**	**68.8195**	**46.042**	**0.9123**	**70.6467**	**47.2913**	**0.9108**	**84.3528**	**56.1575**	**0.9048**
**0.957**	62.5573	41.8519	0.9164	65.2241	43.6754	0.9149	77.3581	51.4858	0.909
**0.911**	56.2407	37.6254	0.9208	58.7655	39.352	0.9192	70.2759	46.7561	0.9134
**0.829**	45.9304	30.7269	0.9284	48.2092	32.2856	0.9269	58.6447	38.9897	0.9212
**0.725**	34.532	23.1007	0.938	36.5113	24.4549	0.9365	45.6465	30.3129	0.9308
**0.625**	25.2545	16.8938	0.947	26.9579	18.0595	0.9455	34.9042	23.1444	0.9399

Significant values are in bold.

## Effects of DGs and EVs on system losses and voltage profile

**Fig. 12:**
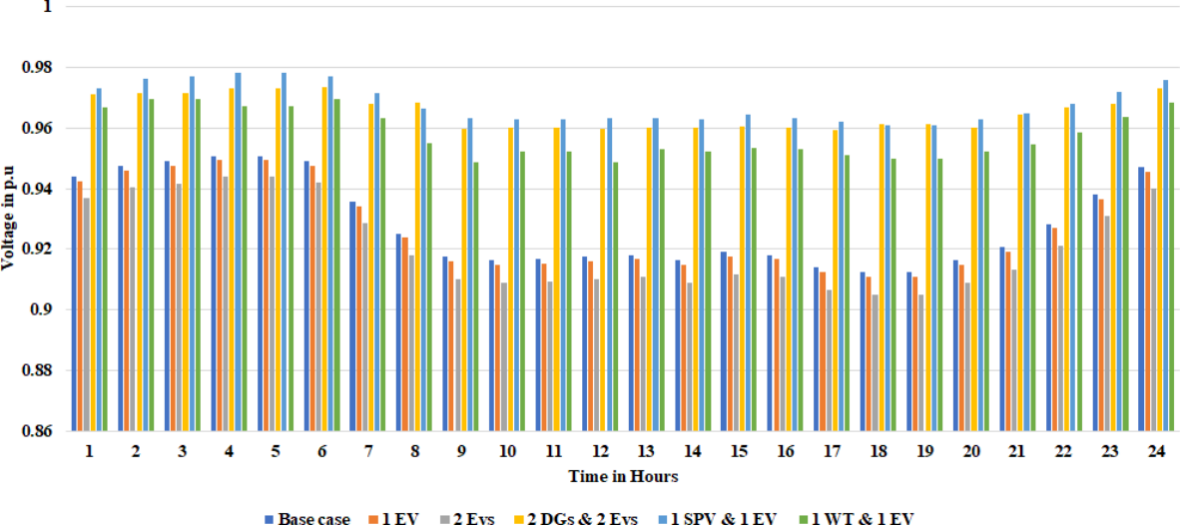
Voltage profile of different scenarios.

**Fig. 13:**
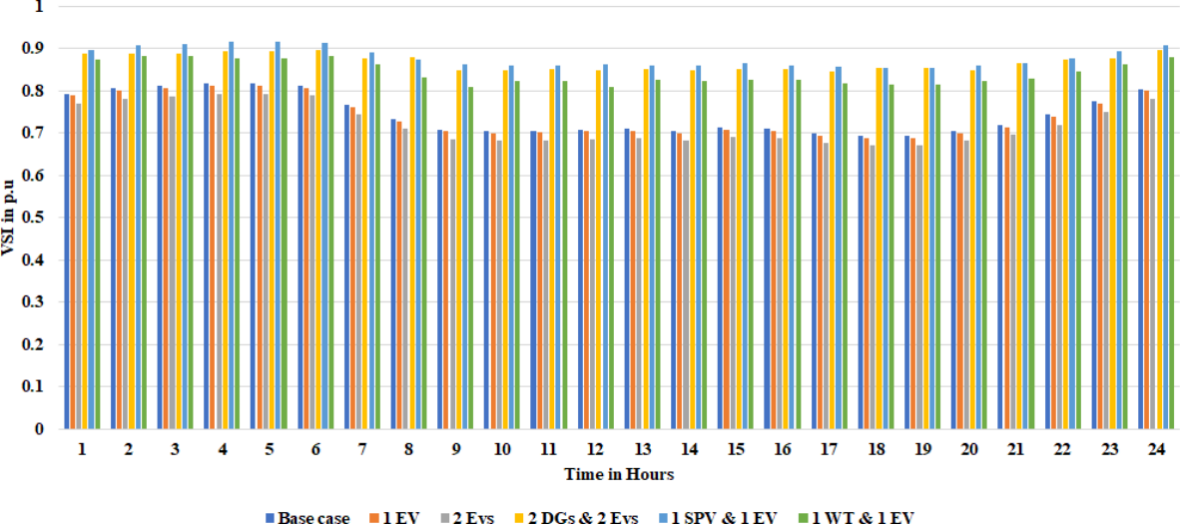
VSI of different scenarios.

**Fig. 14:**
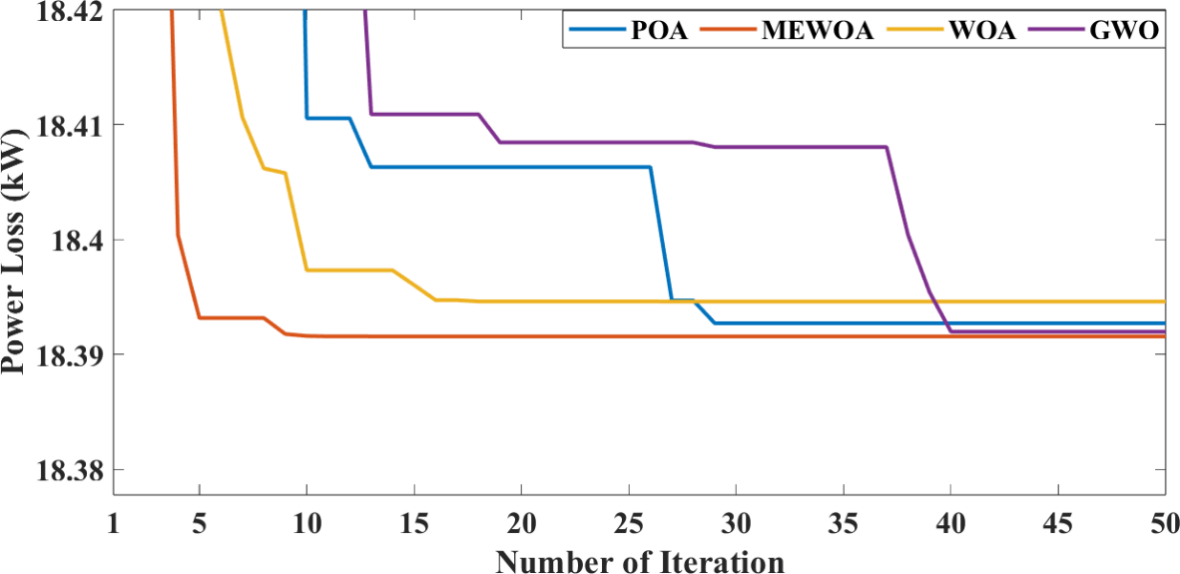
Convergence curve for different algorithms.

**Fig. 15:**
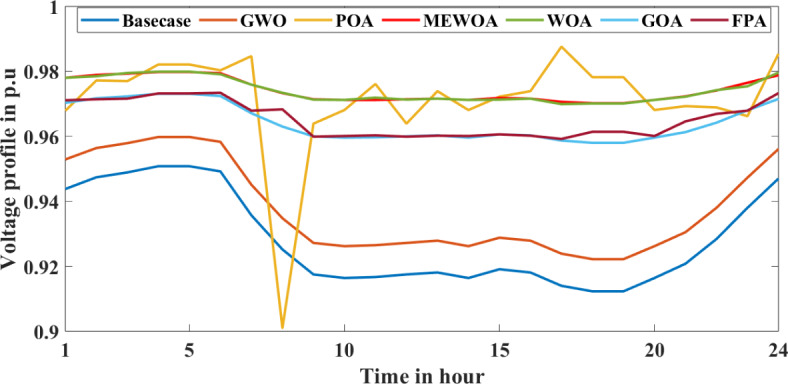
Voltage profile of different algorithms.

With DG integration, there were benefits such as reduced P_Loss_ and improvement. However, with the addition of an EV charging station into RDS, the P_Loss_ increased, and the voltage profile was affected. So, to study the effects of DGs along with EVs, the proposed technique is implemented into the Indian 28 test system. This work uses VSI to determine the optimal location of DG and EV, and the MEWOA technique is used to determine their optimal sizes. We consider the EV charging station to have a fixed rating. Among 60 EVs, this paper considered placing 30 EVs into the two bus locations, 2 and 11. Initially, 30 EVs each, along with PV and WT at different locations, are integrated and simulated results are tabulated in Table 8. From Table 8, as highlighted in bold, the active and reactive P_Loss_ decreases with the integration of two DGs as it compensates for the power consumed by the EVs. Figures 12 and 13 present the voltage profile and VSI under various circumstances. The MEWOA results are compared with six optimisation techniques: GWO, WOA, POA, FPA, and GOA. The parameters of each algorithm are set as follows: search agent = 20, and maximum number of iterations as 50. Table 9 tabulates the simulated results. It is clear from Table 9 that the proposed algorithm provides the best (lower) value of power losses and improved voltage profile. Results of MEWOA are highlighted in bold in Table 9. The convergence curve of different algorithms is shown in Fig. 14. However, there is no convergence curve for FPA, and the GOA convergence curve is omitted as its final values are very high (42.097 kW) for full load demand. Figure 15 depicts the voltage profile of different algorithms.

**Table 8:** Active and reactive P_Loss_ with integration of DGs along with EVs.

	Base Case		1 SPV @7, 1 WT@ 25 and 2 Evs @ 2& 11		1 SPV @ 2 and 1 Evs @ 2		1 WT @ 25 and 1 EV@ 11	
Demand	Active *P*_Loss_	Reactive *P*_Loss_	Active *P*_Loss_	Reactive *P*_Loss_	Active *P*_Loss_	Reactive *P*_Loss_	Active *P*_Loss_	Reactive *P*_Loss_
**0.661**	28.4098	19.0048	19.6041	12.6528	16.3407	10.7543	22.0616	13.7226
**0.621**	24.9165	16.6677	23.7066	15.5503	14.4206	9.4917	19.7322	12.162
**0.604**	23.5078	15.7253	22.8214	14.9577	13.6431	8.9804	18.7236	11.6157
**0.582**	21.7512	14.5501	28.0832	16.2134	12.6709	8.3411	21.4201	14.004
**0.582**	21.7512	14.5501	28.0832	16.2134	12.6709	8.3411	21.4201	14.004
**0.600**	23.1828	15.5079	16.8944	10.8915	13.8271	9.1139	18.5071	11.5099
**0.750**	37.1056	24.8225	24.5518	15.9245	21.075	13.8675	27.9566	17.5657
**0.864**	50.1849	33.5734	79.2909	53.2659	28.0898	18.4801	36.6416	22.7099
**0.946**	61.0112	40.8173	43.391	28.4277	33.8112	22.2426	43.4807	27.2344
**0.957**	62.5573	41.8519	38.6888	25.2058	34.637	22.7874	44.9201	27.7474
**0.954**	62.1334	41.5682	38.5587	25.1158	34.4002	22.6299	44.1994	27.6938
**0.946**	61.0112	40.8173	37.9521	24.7194	33.8112	22.2426	43.4807	27.2344
**0.939**	60.0391	40.1669	37.5109	24.399	33.3007	21.9071	42.9827	27.1468
**0.957**	62.5573	41.8519	38.6888	25.2058	34.637	22.7874	44.9201	27.7474
**0.929**	58.666	39.2481	36.6545	23.8577	44.126	29.3781	41.9735	26.303
**0.939**	60.0391	40.1669	37.5109	24.399	33.3007	21.9071	42.9827	27.1468
**0.982**	66.1555	44.2595	40.6733	26.5051	36.5842	24.0649	46.7704	29.3584
**1.000**	**68.8195**	**46.042**	**43.1486**	**27.9811**	**37.8948**	**24.9279**	**49.6958**	**31.854**
**1.000**	**68.8195**	**46.042**	**43.1486**	**27.9811**	**37.8948**	**24.9279**	**49.6958**	**31.854**
**0.957**	62.5573	41.8519	38.6888	25.2058	34.637	22.7874	44.9201	27.7474
**0.911**	56.2407	37.6254	36.4703	23.7427	32.1734	21.199	40.4111	25.3082
**0.829**	45.9304	30.7269	31.0712	19.8338	25.8208	16.9882	33.7116	21.0934
**0.725**	34.532	23.1007	42.5015	27.9408	19.8689	13.0823	26.2013	16.4078
**0.625**	25.2545	16.8938	18.0552	11.659	14.9321	9.8343	20.1093	12.3023

Significant values are in bold.

**Table 9:** System performance with different algorithms.

Demand	Base case	GWO	POA	MEWOA	WOA	GOA	FPA
**0.661**	28.4098	9.4796	9.4801	**9.4796**	9.4798	19.471	19.6257
**0.621**	24.9165	8.6431	8.6435	**8.643**	8.6512	17.426	17.4325
**0.604**	23.5078	8.3011	8.3014	**8.301**	8.3111	16.5955	16.6468
**0.582**	21.7512	7.8704	7.8712	**7.8704**	7.874	15.5547	15.6316
**0.582**	21.7512	7.8704	7.8712	**7.8704**	7.874	15.5547	15.6316
**0.600**	23.1828	8.2218	8.2217	**8.2217**	8.2325	16.4034	16.4658
**0.750**	37.1056	11.5027	11.5034	**11.5023**	11.5236	24.4842	24.5286
**0.864**	50.1849	14.4215	14.4214	**14.4214**	14.4227	31.8585	33.816
**0.946**	61.0112	16.7515	16.7577	**16.7512**	16.7512	37.8408	37.849
**0.957**	62.5573	17.0787	17.083	**17.0785**	17.0788	38.6873	38.7071
**0.954**	62.1334	16.9893	16.9926	**16.9889**	16.9918	38.4554	38.5082
**0.946**	61.0112	16.7515	16.7577	**16.7512**	16.7512	37.8408	37.849
**0.939**	60.0391	16.5447	16.5453	**16.5447**	16.5504	37.3076	37.5858
**0.957**	62.5573	17.0787	17.083	**17.0785**	17.0788	38.6873	38.7071
**0.929**	58.666	16.253	16.2523	**16.2523**	16.2525	36.5533	36.5579
**0.939**	60.0391	16.5447	16.5453	**16.5447**	16.5504	37.3076	37.5858
**0.982**	66.1555	17.8357	17.8355	**17.8354**	17.8568	40.65	40.6733
**1.000**	68.8195	18.392	18.3982	**18.3916**	18.3991	42.097	43.1486
**1.000**	68.8195	18.392	18.3982	**18.3916**	18.3991	42.097	43.1486
**0.957**	62.5573	17.0787	17.083	**17.0785**	17.0788	38.6873	38.7071
**0.911**	56.2407	15.7331	15.7334	**15.7331**	15.7348	35.217	35.945
**0.829**	45.9304	13.486	13.5276	**13.4858**	13.4858	29.479	30.1505
**0.725**	34.532	10.9117	10.9118	**10.9116**	10.9163	23.0109	23.0112
**0.625**	25.2545	8.7247	8.7273	**8.7246**	8.7253	17.6248	17.905

Significant values are in bold.

## Conclusion

This paper discusses identifying the weak and strong buses using the VSI for the Indian 28 test system and the optimal allocation of the general RDGs and Solar PV and WT considering uncertainty. The MEWOA algorithm has been adopted to allocate the DGs and EVs optimally. The Beta and Weibull probability distribution functions are employed to model the uncertainties of the Solar PV and WT DGs. The distribution system is integrated with different scenarios considering the combination of DGs, Solar PV, WT, and EVs at various bus locations. The simulated results show that the power losses decrease when the single or double DGs are integrated into RDS. However, when the EVs with fixed ratings are connected to the RDS, the power losses increase slightly depending upon the rating of the EV charging stations. To harness the optimum benefits from the RDGs, DGs and EVs are integrated simultaneously into the RDS, obtaining the optimal power losses and improved voltage profile. In addition, the effects of uncertainties of solar PV and WT are studied in this paper. Uncertainties of solar PV and WT are generated for 24 h for a year. Due to dependency on solar irradiance and wind speed, the power outputs vary throughout the day. However, with uncertainty, the planning engineers will benefit precisely during load generation and planning process forecasting. The future scope of this work, related to different types of EVs and its charging and discharging patterns and driving patterns, needs to be considered for better assessment.

## Electronic supplementary material

Below is the link to the electronic supplementary material.

Supplementary Material 1

## Data Availability

The datasets used and/or analysed during the current study are available from the corresponding author upon reasonable request.
