# Supplementary material for: Optimal placement of renewable distributed generators and electric vehicles using multi-population evolution whale optimization algorithm
Source: Sci Rep. 2024 Nov 18;14:28447. doi: 10.1038/s41598-024-80076-z (PMC11574299; doi:10.1038/s41598-024-80076-z)
Supplement: Supplementary file 1 — Supplementary Material 1 [file 41598_2024_80076_MOESM1_ESM.docx]

**Nomenclature:**

MEWOA Multi-population Evolution Whale Optimization Algorithm

DGs Distributed Generators

WT Wind Turbine

SPV Solar Photovoltaic

RDS Radial Distribution System

EV Electric Vehicles

P_Loss_ Power Loss

RES Renewable Energy Resources

EVCS Electric Vehicles Charging Station

PDF Probability Distribution Function

VSI Voltage Stability Index

α, β Shape parameter of Beta PDF

Γ Gamma Function

S Solar irradiance (kW/m^2^)

μ Mean

σ Standard Deviation

FF Fill factor

𝑇_𝐴_ Ambient temperature (°C)

$T_{cn}$ Cell Temperature at nth states

$K_{v}$ Voltage temperature coefficient (V/°C)

$K_{i}$ Current temperature co-efficient (A/°C)

$V_{MPP}$ Voltage at Maximum power point (V)

$I_{MPP}$ Current at Maximum power point (A)

$N_{OT}$ Normal Operating temperature of a cell (°C)

$V_{OC}$ Open circuit voltage (V) at maximum power point.

$I_{SC}$ Short Circuit Current at Maximum power point

MPP Maximum power point

𝑇𝐸𝑃𝑂 Total Expected power output

K Shape parameter of WT

C Scale factor of WT

$P_{WT}$ Power Output of WT

P(S) Power Output of Solar PV

${SoC}^{min}$ State of Charge minimum of EV

${SoC}^{max}$ State of Charge maximum of EV

t Number of trips of EV

d Distance travelled by EV

$d_{c}$ Distance covered by EV in range

$P_{G}^{SS}$ Power generated from grid substation

$P_{DG}^{PV}$ Power generated by Solar PV DGs

$P_{DG}^{WT}$ Power generated by WT DGs

$P_{Demand}$ Total Demand of the systems

$P_{EV}$ Power consumed by EV while charging

WOA Whale Optimisation Algorithm

$X^{rand}$ Random Whale population

lb Lower bound

ub Upper bound

DLF Distribution Load Flow

RDGs Renewable Distributed Generators

kW Kilo Watts

kVAr Kilo Volt Ampere Reactive

DN Distribution Network
